# 509. *Pseudomonas aeruginosa*-specific CRISPR-Cas3 bacteriophage cocktail developed to treat respiratory and bloodstream infections

**DOI:** 10.1093/ofid/ofae631.161

**Published:** 2025-01-29

**Authors:** Lana McMillan, Taylor Penke, Hannah Tuson, Robert McKee, Ethan Baker, Ashley Trama

**Affiliations:** Locus Biosciences, Morrisville, North Carolina; Locus Biosciences, Morrisville, North Carolina; Locus Biosciences, Morrisville, North Carolina; Locus Biosciences, Morrisville, North Carolina; Locus Biosciences, Morrisville, North Carolina; Locus Biosciences, Morrisville, North Carolina

## Abstract

**Background:**

New strategies are needed to combat infections due to multidrug-resistant (MDR) Pseudomonas aeruginosa. Bacteriophage therapy offers an effective, pathogen-specific, microbiome-sparing treatment with an orthogonal mechanism of action to that of small-molecule antibiotics. Here we describe the development and characterization of the *P. aeruginosa*-targeting 6-phage cocktail LBP-PA01.

**Methods:**

High-throughput automated methods were used to isolate and characterize wild-type phages. Proprietary Locus-developed software was used to predict the most efficacious combinations of bacteriophages. Selected phages were engineered to express CRISPR-Cas3, and phages were combined into an optimal cocktail and subjected to *in vitro* antibacterial activity characterization including assessments of bacteriophage and cocktail host range by liquid growth curve assay and by plaque assay, bactericidal activity in the presence of mucin matrices using EpiAirway^TM^ human airway epithelium tissues, biofilm degradation and inhibition. *In vivo* efficacy was tested in immuno- compentent mouse models of lower respiratory tract infection with LBP-PA01 administered intranasally and intravenously as well as in a rat model of blood stream infection with intravenous administration.

**Results:**

>2800 individual bacteriophage were discovered through high throughput methods. Cocktail prediction and optimization resulted in the final combination of 6 unique bacteriophage, LBP-PA01, which was tested against 570 diverse contemporary *P. aeruginosa* clinical isolates from patients with respiratory or bloodstream infections. The cocktail showed a host range of 97.3% and efficacy against biofilms and in the presence of mucin. In mouse models of acute lower respiratory tract infection and disseminating pulmonary infection and a rat model of bloodstream infection, LBP-PA01 produced multiple log reductions in CFU levels relative to vehicle treatment and superiority to standard-of-care antibiotics.

**Conclusion:**

Based on the in vitro and mouse model data, LBP-PA01 is a strong candidate for advancement into the clinic for treatment for *P. aeruginosa* infections.

**Disclosures:**

**All Authors**: No reported disclosures

